# Effect of Estrogen Receptor on the Relationship Between HER2 Immunohistochemistry Score and Pathological Complete Response to Neoadjuvant Treatment in HER2-Positive Breast Cancer

**DOI:** 10.1155/2024/8851703

**Published:** 2024-10-10

**Authors:** Miaomiao Jia, Haibo Yang, Lihui Pan, Jinnan Gao, Fan Guo

**Affiliations:** Department of Breast Surgery, Shanxi Bethune Hospital, Shanxi Academy of Medical Sciences, Tongji Shanxi Hospital, Third Hospital of Shanxi Medical University, Taiyuan 030032, China

**Keywords:** breast cancer, estrogen receptor, human epidermal growth factor receptor 2, pathological complete response

## Abstract

**Purpose:** We aimed to investigate whether estrogen receptor (ER) status affects the predictive role of the human epidermal growth factor receptor 2 (HER2) immunohistochemistry (IHC) score on the efficacy of neoadjuvant treatment for HER2-positive breast cancer.

**Methods:** This retrospective study comprised 167 individuals diagnosed with HER2-positive invasive breast cancer who had undergone neoadjuvant treatment and surgery. Uni- and multivariable logistic regression analyses were performed on the relationship between the HER2 IHC score and total pathological complete response (tpCR), breast pathological complete response (bpCR), or axillary partial response (apCR). Subgroup analyses were used to investigate whether the relationship between the HER2 IHC score and tpCR, bpCR, or apCR differed by ER or PR status.

**Results:** The overall tpCR rate for HER2-positive breast cancers treated with neoadjuvant treatment was 41.32% (69 of 167). The tpCR, bpCR, and apCR rates were greater in the HER2 IHC 3+ group (tpCR: IHC 3 + 47.69% vs. IHC 2 + 18.92%, *p*=0.009). Significant interactions between HER2 IHC score and tpCR or bpCR were found in subgroup analyses based on ER status (tpCR: *p* for interaction = 0.001; bpCR: *p* for interaction = 0.001). Among ER-positive patients, the HER2 IHC 2+ group had substantially decreased tpCR, bpCR, and apCR rates than the HER2 IHC 3+ group (tpCR rate: *p*=0.003; bpCR rate: *p*=0.002; apCR rate: *p*=0.002). For ER-negative individuals, the tpCR, bpCR, and apCR rates did not differ significantly among the HER2 IHC 3+ versus HER2 IHC 2+ groups. Similarly, interactions between HER2 IHC score and tpCR, bpCR, or apCR were found in subgroup analyses based on PR status.

**Conclusion:** HER2 IHC 2+ may indicate a decreased tpCR rate, bpCR rate, and apCR rate to neoadjuvant treatment in HR-positive patients having HER2-positive breast cancer, but not in HR-negative patients.

## 1. Introduction

The proportion of human epidermal growth factor receptor 2 (HER2)-positive initial breast cancers is believed to be between 15% and 20% [1]. Metastasis to the viscera and central nervous system is common in HER2-positive breast cancer, further worsening the prognosis [2]. Neoadjuvant treatment is an important treatment modality for early-stage HER2-positive breast cancer. It not only shrinks the primary tumor and provides patients with the opportunity for surgical, breast-conserving, or omission of axillary lymph node dissection, but also evaluates the efficacy of the medications, predicts the prognosis, and screens out treatment-naïve patients who would benefit from intensive treatment [3, 4]. Achievement of pathological complete response (pCR) after neoadjuvant chemotherapy results in improved rates of disease-free survival (DFS) and overall survival (OS), and is an important evaluation index of the effectiveness of neoadjuvant treatment [5, 6]. Axillary pCR has been reported to be associated with improved 10-year OS and relapse-free survival (RFS) [7]. Neoadjuvant chemotherapy combined with HER2-targeted therapy is now the standard neoadjuvant treatment regimen for HER2-positive breast cancer, and it has been shown to increase the pCR rate [8–12]. However, there are still many patients who do not achieve a pCR. Therefore, it is crucial to identify the factors that influence pCR to neoadjuvant treatment for HER2-positive breast cancer. This will enable us to adjust the treatment regimen accordingly.

Immunohistochemistry (IHC) and fluorescence in situ hybridization (FISH) are two procedures commonly used for determining HER2 status. The current American Society of Clinical Oncology/College of American Pathologists (ASCO/CAP) criteria define HER2 positivity when the IHC score is 3+ or 2+ and FISH amplification is evident. HER2 IHC score has been reported to be a predictor of pCR after neoadjuvant treatment for HER2-positive breast cancer. Compared with HER2 IHC 2+ patients, HER2 IHC 3+ patients have a higher pCR rate [13–16]. In addition, several studies have shown that hormone receptor (HR)-positive patients with HER2-positive breast cancer are less sensitive to neoadjuvant chemotherapy plus HER2-targeted therapy than HR-negative patients, resulting in lower pCR rates and poorer DFS and OS [12, 17–21]. The signaling pathways involving the estrogen receptor (ER) and HER2 have been demonstrated to interact with each other [22]. Whether ER status affects the relationship between HER2 IHC score and pCR to neoadjuvant treatment has not been established.

The main clinical aims of neoadjuvant treatment are to downstage tumor stage, achieve breast conservation, and omit axillary lymph node dissection. In this retrospective study, we assessed whether the HER2 IHC score could accurately predict the efficacy of neoadjuvant treatment in HER2-positive breast cancers, such as total pathological complete response (tpCR), breast pathological complete response (bopper), and axillary pathological complete response (apCR). The study also examined whether there was a difference in the relationship between HER2 IHC score and the efficacy of neoadjuvant treatment depending on the ER or progesterone receptor (PR) status of the patients. This may provide a scientific basis for the individualized development or adjustment of neoadjuvant treatment regimens for HER2-positive breast cancer patients.

## 2. Materials and Methods

### 2.1. Study Population

We retrospectively collected patients who were diagnosed with HER2-positive invasive breast cancer and underwent neoadjuvant therapy and surgery between March 2012 and March 2023 at Shanxi Bethune Hospital. Patients with tumors larger than 2 cm or with axillary lymph node involvement were chosen for neoadjuvant treatment [6, 23, 24]. The following were the criteria for inclusion: newly diagnosed invasive breast cancer by core needle biopsy; HER2-positive; treated at Shanxi Bethune Hospital initially; experiencing neoadjuvant treatment, which might consist of chemotherapy alone, chemotherapy along with trastuzumab, or chemotherapy coupled with both trastuzumab and pertuzumab; and undergoing surgery and postoperative pathological evaluation after neoadjuvant treatment. Male patients, patients with metastatic illness, patients with a history of malignancy, patients with bilateral breast cancer, and patients without clinicopathological data were not included. For this research, information about age at diagnosis, body mass index (BMI), tumor and lymph node stages, ER and PR status, HER2 IHC score, Ki67 index, neoadjuvant chemotherapy regimen, HER2-targeted treatment regimen, and postoperative pathology was collected.

### 2.2. Definitions

The efficacy of neoadjuvant treatment was assessed based on postoperative pathological findings. When there is no detectable invasive cancer in the breast or the ipsilateral axillary lymph nodes, regardless of residual ductal carcinoma in situ (ypT0/TisN0M0), tpCR could be diagnosed. The absence of residual invasive cancer in the breast, regardless of residual ductal carcinoma in situ (ypT0/Tis), is called a bpCR. When there is no evidence of cancer in the ipsilateral axillary lymph nodes (ypN0), this is referred to as an axillary pathologic complete response (apCR) [25, 26].

All patients underwent breast physical examination, breast ultrasound, and mammography. An ultrasound-guided core needle biopsy was used to obtain focal breast tissue for pathological confirmation. The seventh edition of the breast cancer staging system developed by the American Joint Committee on Cancer (AJCC) was implemented to determine the clinical T stage and the clinical N stage of the patient. Before neoadjuvant therapy, we performed core needle biopsies of axillary lymph nodes suspected to be involved in physical examination or imaging. Pathology confirmed the diagnosis of axillary lymph node involvement. The expression of HER2, ER, and PR were calculated using the CAP/ASCO criteria. ER or PR-positive staining was defined as more than 1% of tumor nuclei staining with ER or PR antibodies. HR-positive was defined as ER-positive and/or PR-positive. HER2-positive was defined as a HER2 IHC score of 3+ or 2+/FISH-amplified. In > 10% of tumor cells, strong complete membrane staining was seen, resulting in an IHC score of 3+. An IHC score of 2+ was indicated by weak or moderate complete membrane staining in > 10% of tumor cells. It was also defined as strong complete membrane staining in ≤ 10% of tumor cells. The HER2 gene and CEP17 chromosomal status were further evaluated by FISH if the IHC score was 2+. A HER2/CEP17 ratio of ≥2.0 or an average HER2 copy number of ≥ 6.0 was considered FISH amplification.

### 2.3. Statistical Analyses

There was a comparison of clinicopathological features between the groups. The mean and standard deviation (*x* ± *s*) were applied to characterize the continuous variables that fit a normal distribution. We utilized the Student's *t*-tests to compare means, whereas the median (interquartile range) [M (P25, P75)] was used to depict and the Mann-Whitney *U* tests were then performed to evaluate data that did not follow a normal distribution. The *n* and percentage of instances were used to characterize the categorical variables. We employed the chi-square test or Fisher's precise probability approach.

A univariate logistic regression evaluation was performed to find possible predictors of pCR (*p* < 0.1). Adjusting for covariates with *p* < 0.1 in univariate logistic regression, we performed multivariable logistic regression analyses to examine the effect of HER2 IHC score on tpCR, bpCR, and apCR. The impact of HER2 IHC score on tpCR, bpCR, and apCR across varying ER and PR statuses was examined using subgroup analysis. The likelihood ratio was used to evaluate the potential impact of subgroup interactions. Forest plots were drawn for visualization.

All statistical tests were two-sided, and differences were considered statistically significant at *p* < 0.05.

## 3. Results

### 3.1. Patient Characteristics

**Figure 1:**
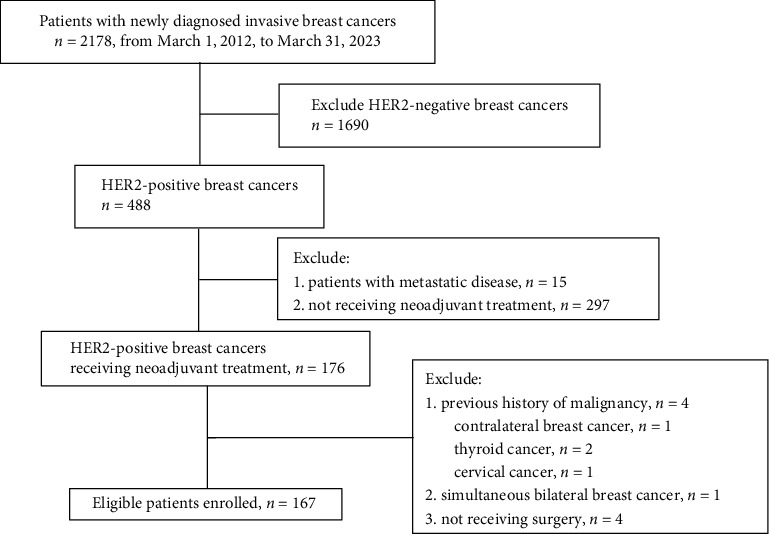
Flowchart of patient screening.

A total of 2178 newly diagnosed invasive breast cancer patients were admitted from March 1, 2012, to March 31, 2023. Of those, 488 (22.41%) had HER2-positive breast cancer. This study eventually included 167 patients (Figure 1). The clinicopathological characteristics of all patients are presented in Table 1. Among the included patients, 130 presented with HER2 IHC 3+, and 37 presented with IHC 2+/FISH-amplified. All patients received taxane-based chemotherapy treatment. The majority of patients (90.42%) were given HER2-targeted treatment, and over half (58.08%) were given dual HER2-targeted treatment. In total, 58.08% (97/167) of patients showed ER positivity, and 42.51% (71/167) exhibited PR positivity. The initial clinical T stage was predominantly T2 (64.07%), with axillary lymph node involvement in 89.82% (150/167) of patients. ER positivity was common among HER2 IHC 2+ patients (28/37, 75.68%). Patients with HER2 IHC 3+ were more likely to undergo TCb (taxane + carboplatin) chemotherapy regimens.

### 3.2. Prediction of pCR by HER2 IHC Score

After neoadjuvant therapy, the overall tpCR rate was 41.32% (69/167), the bpCR rate was 44.31% (74/167), and the apCR rate was 59.28% (99/167). Among HER2 IHC 3+ patients, 47.69% (62/130) achieved a tpCR, but only 18.92% (7/37) did among IHC 2+ patients. Additionally, HER2 3+ patients had a greater bpCR rate (51.54% vs. 18.92%) and an apCR rate (66.15% vs. 35.14%) than HER2 2+ patients.

Using uni- and multivariable logistic regression, results examining the relationship between HER2 IHC score and tpCR are displayed in Table 2. The tpCR rate was notably higher in HER2 IHC 3+ patients compared with HER2 IHC 2+ patients [odds ratio (OR), 3.772; 95% confidence interval (95% CI), (1.466, 10.869); *p*=0.009]. The incidence of bpCR and apCR was also considerably higher in IHC 3+ patients than in IHC 2+ individuals (bpCR: OR, 4.329; *p*=0.003; apCR: OR, 3.255; *p*=0.006).

### 3.3. Prediction of pCR by HER2 IHC in Different ER and PR Statuses

**Figure 2:**
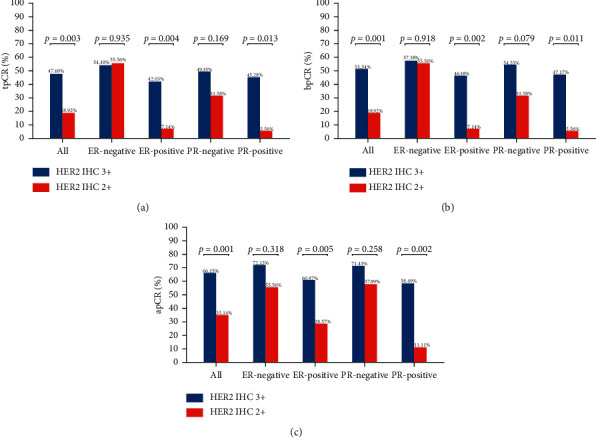
The tpCR rate (a), bpCR rate (b), and apCR rate (c) in different ER and PR statuses according to HER2 IHC score.

Figure 2 depicts the tpCR rates for individuals with varying ER and PR statuses who tested positive for HER2 (IHC 2+ or IHC 3+). Whether ER-positive or PR-positive, patients with HER2 IHC 2+ had a lower tpCR rate than those with HER2 IHC 3+ (tpCR rate in ER-positive patients: IHC 2 + 7.14% vs. IHC 3 + 42.03%; *p*=0.004) (tpCR rate in PR-positive patients: IHC 2 + 5.56% vs. IHC 3 + 45.28%; *p*=0.013). IHC 2+ individuals also had a considerably decreased bpCR rate and apCR rate. Nevertheless, HER2 IHC 3+ and HER2 IHC 2+ individuals who were ER-negative or PR-negative showed not a statistically significant disparity in tpCR, bpCR, or apCR rates (tpCR rate in ER-negative patients: IHC 2 + 55.56% vs. IHC 3 + 54.10%; *p*=0.935) (tpCR rate in PR-negative patients: IHC 2 + 31.58% vs. IHC 3 + 49.35%; *p*=0.169).

**Figure 3:**
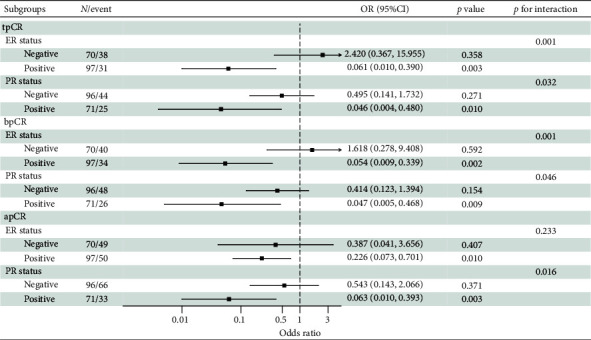
Subgroup analyses stratified by ER and PR status comparing the tpCR rate, bpCR rate, and apCR rate between HER2 IHC 2+ and IHC 3+ patients.

Based on these findings, we conducted subgroup analyses (Figure 3). In analyses stratified by ER status, significant interactions between HER2 IHC score and tpCR or bpCR were observed (tpCR: *p* for interaction = 0.001; bpCR: *p* for interaction = 0.001). The same results were observed stratified by PR status. Lower rates of tpCR [ER-positive: OR, 0.061; 95% CI (0.010, 0.390), *p*=0.003; PR-positive: OR, 0.046; 95% CI (0.004, 0.480), *p*=0.010], bpCR [ER-positive: OR, 0.054; 95% CI (0.009, 0.339), *p*=0.002; PR-positive: OR, 0.047; 95% CI (0.005, 0.468), *p*=0.009], and apCR [ER-positive: OR, 0.226; 95% CI (0.073, 0.701), *p*=0.010; PR positive: OR, 0.063; 95% CI (0.010, 0.393), *p*=0.003] were predicted when HER2 IHC score was 2+. Neither the tpCR rate, the bpCR rate, nor the apCR rate for ER-negative or PR-negative patients was affected by the HER2 IHC score (*p* > 0.05).

## 4. Discussion

This study aimed to investigate the hypothesis that the presence or absence of ER affects the predictive value of HER2 IHC score on pCR to neoadjuvant treatment in HER2-positive breast cancer patients.

As mentioned in previous studies, the HER2 IHC score may be an effective predictive factor for the efficacy of neoadjuvant treatment for HER2-positive breast cancer. In the present study, the tpCR rate, bpCR rate, and apCR rate after neoadjuvant treatment were significantly higher in IHC 3+ patients than in IHC 2+ patients. A recent study showed that HER2 3+ tumors were significantly more likely to achieve tpCR (IHC 3+ 55.1% vs. IHC 2+ 17.6%, *p* < 0.001) [27]. The same results were found in Bin's study, with tpCR rates of 47.86% and 22.22%, respectively, in the IHC 3+ and IHC 2+ groups using either mono- or dual-targeted treatment [15]. Additionally, Ayaka also found similar results with tpCR rates of 51.8% and 20.3%, respectively [28]. Almost all studies on the effect of HER2 IHC score on pCR after neoadjuvant treatment focused on the outcome of tpCR. Our study also showed that the bpCR rate and apCR rate after neoadjuvant treatment were significantly higher in IHC 3+ patients than in IHC 2+ patients. Trastuzumab-mediated antibody-dependent cytotoxicity (ADCC) is one of its antitumor mechanisms, and its effect correlates with HER2 expression levels [29]. Accordingly, the HER2 IHC score and HER2-targeted treatment efficacy are positively correlated. In our study, 90.42% of patients received HER2-targeted treatment. Our study confirms previous findings that neoadjuvant treatments are more effective for HER2 IHC 3+ patients.

Additionally, this study showed a lower tpCR rate in HER2-positive/ER-positive patients than in HER2-positive/ER-negative patients (31.96% vs. 54.29%). PR status had the same effect on tpCR. As evidenced by the NeoSphere trial and the PEONY trial, the neoadjuvant population receiving chemotherapy in combination with dual-targeted treatment exhibited tpCR rates of 26.0% and 33.3% in HER2-positive/HR-positive patients and 63.2% and 46.1% in HER2-positive/HR-negative patients [9, 10]. Another pooled analysis including 12 studies confirmed a tpCR rate of 30.9% for HER2-positive/HR-positive breast cancer in the presence of trastuzumab combined with chemotherapy, which was significantly lower than the 50.3% rate for HER2-positive/HR-negative patients [21]. Overall, HR positivity affects the efficacy of HER2-positive breast cancer to neoadjuvant treatment, leading to lower tpCR rates.

Previously, HER2 IHC 3+ and HER2 IHC 2+/FISH-amplified tumors have been reported to have different pathological characteristics, with IHC 2+ tumors usually being HR-positive [15, 27, 30]. In our study, 75.68% of HER2 IHC 2+ patients were ER-positive, showing a higher percentage than HER2 IHC 3+ patients (53.08%). Yan et al. suggested that the high proportion of ER positivity in the IHC 2+ group may account for the low tpCR rate in HER2 IHC 2+ breast cancers [13]. Moreover, we observed the interaction between HER2 IHC score and tpCR under different ER or PR statuses. However, this interaction has not previously been determined. Subgroup analyses based on ER and PR status suggested no significant impact of the HER2 IHC score on tpCR in HER2-positive/ER-negative or HER2-positive/PR-negative patients. However, the HER2 IHC score positively correlates with tpCR in HER2-positive/ER- or PR-positive patients, with the HER2 IHC 2+/ER- or PR-positive subgroup showing the poorest tpCR benefit. These findings were similar to the results from a previously reported study. In that study, ER status independently predicted tpCR in patients with IHC 2+/FISH-amplified tumors, with a tpCR rate of only 15% in the HER2 IHC 2+/ER-positive group, which was significantly lower than that in the HER2 IHC 2+/ER-negative group (36% vs. 15%, *p*=0.02) [28]. In another study, however, HER2 IHC score was highly correlated with tpCR, regardless of whether in HR-negative or HR-positive patients [31]. It might be because patients in the study received dual-targeted treatment with lapatinib and trastuzumab, which was different from the HER2-targeted regimen in our study. Overall, this study revealed that the HER2 IHC score predicted pCR to neoadjuvant treatment in HER2-positive breast cancer patients. This phenomenon only occurs in ER- or PR-positive individuals rather than ER- or PR-negative individuals.

These results emphasize the necessity to evaluate both HER2 IHC score and HR status together to identify individuals with the poorest benefit from neoadjuvant treatment. However, it must be stressed that this research has a few limitations. First, this was a retrospective, non-randomized study. Second, as a result of the small sample size, some subgroup analyses had limited and potentially biased results, particularly in the HER2 IHC 2+/ER-negative population (*n* = 9). Third, not all HER2-positive patients in our sample were given HER2-targeted therapy. Finally, survival analyses were not performed due to the short follow-up of most patients in our study, particularly those who were given dual HER2-targeted drugs. Further follow-up or large-scale studies are necessary to observe whether patients in the HR-positive group had improved survival compared with HR-negative patients, despite their lesser response to neoadjuvant treatment. As a result, the results need to be interpreted with caution. Additional large-scale, multicenter trials of patients undergoing dual HER2-targeted therapy are needed.

## 5. Conclusion

The presence or absence of HR has been found to affect the relationship between HER2 IHC score and pCR to neoadjuvant treatment in HER2-positive breast cancer patients. In HR-positive patients, the HER2 IHC 2+ group had considerably lower tpCR, bpCR, and apCR rates than the HER2 IHC 3+ group. However, the HER2 IHC score did not affect tpCR, bpCR, and apCR in HR-negative patients. HER2 IHC 2+/HR-positive breast cancer patients may require more attention as well as additional therapeutic approaches to improve tpCR rates and clinical outcomes.

## Figures and Tables

**Table 1:** Clinicopathological characteristics stratified by HER2 IHC score (IHC 3+ vs. IHC 2+).

**Characteristic**	**All patients**	**HER2 IHC 2+**	**HER2 IHC 3+**	**p** ** value**
	**(*n* = 167)**	**(*n* = 37)**	**(*n* = 130)**	
Age, mean ± SD, y	48.40 ± 10.12	50.62 ± 9.93	47.77 ± 10.13	0.130
BMI, mean ± SD	24.16 ± 3.44	24.53 ± 3.85	24.05 ± 3.33	0.497
Menopausal status				0.560
Premenopausal	95 (56.89%)	19 (51.35%)	76 (58.46%)	
Postmenopausal	72 (43.11%)	18 (48.65%)	54 (41.54%)	
Clinical T stage				0.131
T1	25 (14.97%)	5 (13.51%)	20 (15.38%)	
T2	107 (64.07%)	24 (64.86%)	83 (63.85%)	
T3	25 (14.97%)	3 (8.11%)	22 (16.92%)	
T4	10 (5.99%)	5 (13.51%)	5 (3.85%)	
Clinical N stage				0.672
N0	17 (10.18%)	3 (8.11%)	14 (10.77%)	
N1	78 (46.71%)	16 (43.24%)	62 (47.69%)	
N2	37 (22.16%)	11 (29.73%)	26 (20.00%)	
N3	35 (20.96%)	7 (18.92%)	28 (21.54%)	
ER status				0.023
Negative	70 (41.92%)	9 (24.32%)	61 (46.92%)	
Positive	97 (58.08%)	28 (75.68%)	69 (53.08%)	
PR status				0.505
Negative	96 (57.49%)	19 (51.35%)	77 (59.23%)	
Positive	71 (42.51%)	18 (48.65%)	53 (40.77%)	
Ki67 index, median (IQR)	40.00 [30.00, 60.00]	50.00 [40.00, 60.00]	40.00 [30.00, 60.00]	0.441
HER2-targeted therapy				0.961
None	16 (9.58%)	4 (10.81%)	12 (9.23%)	
Trastuzumab alone	54 (32.34%)	12 (32.43%)	42 (32.31%)	
Trastuzumab and pertuzumab	97 (58.08%)	21 (56.76%)	76 (58.46%)	
Chemotherapy regimen				<0.001
EC-T^∗^	72 (43.11%)	27 (72.97%)	45 (34.62%)	
TE	6 (3.59%)	0 (0.00%)	6 (4.62%)	
T^∗^	7 (4.19%)	1 (2.70%)	6 (4.62%)	
T^∗^Cb	82 (49.10%)	9 (24.32%)	73 (56.15%)	
tpCR				0.003
No	98 (58.68%)	30 (81.08%)	68 (52.31%)	
Yes	69 (41.32%)	7 (18.92%)	62 (47.69%)	
bpCR				0.001
No	93 (55.69%)	30 (81.08%)	63 (48.46%)	
Yes	74 (44.31%)	7 (18.92%)	67 (51.54%)	
apCR				0.001
No	68 (40.72%)	24 (64.86%)	44 (33.85%)	
Yes	99 (59.28%)	13 (35.14%)	86 (66.15%)	

Abbreviations: apCR = axillary pCR, BMI = body mass index, bpCR = breast pCR, CI = confidence interval, EC-T^∗^ anthracycline + cyclophosphamide sequentially taxane, ER = estrogen receptor, HER2 = human epidermal growth factor receptor 2, IQR = interquartile range, IHC = immunohistochemistry, N = node, pCR = pathological complete response, PR = progesterone receptor, SD = standard deviation, T = tumor, T^∗^ = taxane, TCb = taxane + carboplatin, T^∗^E = taxane + anthracycline, tpCR = total pCR.

**Table 2:** Differences in response to neoadjuvant therapy in patients with HER2 IHC 3+ compared to those with HER2 IHC 2+.

**Outcome**	**Univariate analysis**		**Multivariate analysis**	
	**Odds ratio (95% CI)**	**p** ** value**	**Odds ratio (95% CI)**	**p** ** value**
tpCR	3.908 (1.682, 10.253)	0.003	3.772 (1.466, 10.869)	0.009
bpCR	4.558 (1.963, 11.961)	0.001	4.329 (1.721, 12.218)	0.003
apCR	3.608 (1.701, 7.956)	0.001	3.255 (1.409, 7.779)	0.006

Abbreviations: apCR = axillary pCR, bpCR = breast pCR, CI = confidence interval, HER2 = human epidermal growth factor receptor 2, IHC = immunohistochemistry, pCR = pathological complete response, tpCR = total pCR.

## Data Availability

The datasets generated during and/or analyzed during the current study are available from the corresponding author upon reasonable request.
